# Integrated Genomic Analysis of Sézary Syndrome

**DOI:** 10.4061/2011/980150

**Published:** 2011-11-24

**Authors:** Xin Mao, Tracy Chaplin, Bryan D. Young

**Affiliations:** ^1^Centre for Cutaneous Research, Institute of Cell and Molecular Sciences, Barts and The London School of Medicine and Dentistry, London E1 2AT, UK; ^2^Division of Investigative Science, Department of Histopathology, Faculty of Medicine, Imperial College, Hammersmith Hospital, Du cane Road, London W12 0NN, UK; ^3^Cancer Research UK Medical Oncology Centre, Barts and The London School of Medicine and Dentistry, Queen Mary College, Queen Mary University of London, London EC1M 6BQ, UK

## Abstract

Sézary syndrome (SS) is a rare variant of primary cutaneous T-cell lymphoma. Little is known about the underlying pathogenesis of S. To address this issue, we used Affymetrix 10K SNP microarray to analyse 13 DNA samples isolated from 8 SS patients and qPCR with ABI TaqMan SNP genotyping assays for the validation of the SNP microarray results. In addition, we tested the impact of SNP loss of heterozygosity (LOH) identified in SS cases on the gene expression profiles of SS cases detected with Affymetrix GeneChip U133A. The results showed: (1) frequent SNP copy number change and LOH involving 1, 2p, 3, 4q, 5q, 6, 7p, 8, 9, 10, 11, 12q, 13, 14, 16q, 17, and 20, (2) reduced SNP copy number at FAT gene (4q35) in 75% of SS cases, and (3) the separation of all SS cases from normal control samples by SNP LOH gene clusters at chromosome regions of 9q31q34, 10p11q26, and 13q11q12. These findings provide some intriguing information for our current understanding of the molecular pathogenesis of this tumour and suggest the possibility of presence of functional SNP LOH in SS tumour cells.

## 1. Introduction

Sézary syndrome (SS) is a rare subset of primary cutaneous T-cell lymphoma (CTCL) with an aggressive clinical course [1, 2]. SS typically presents with generalised skin lesions or erythroderma, intense pruritus, and peripheral lymphadenopathy. A blood test will reveal atypical T-lymphocytes with convoluted nuclei, Sézary cells, which are often associated with a cutaneous T-cell lymphoma [2–5]. Recent studies have revealed complex genetic aberrations affecting almost all chromosomes [6–14] with loss of heterozygosity (LOH), [9, 15–19] and deregulation of several genes, [20–26] and epigenetic changes [27, 28] in SS. Despite this progress, the key molecular targets underlying the pathogenesis of this type of skin lymphoma remain elusive. 

Single nucleotide polymorphism (SNP) denotes a single base in the DNA sequence that differs from the usual base at that position. Millions of SNPs have been catalogued in the human genome, which may be responsible for disease such as cancer (http://www.ncbi.nlm.nih.gov/sites/entrez?db=snp). SNP microarray is a powerful genome research technique designed for the identification of SNP and dose change within the whole genome [29–31]. This method has been widely used to investigate SNP copy number change and LOH in cancer genome in a variety of medical and biological subjects [32–42]. However, there has been no report describing the use of SNP microarray to assess genomewide SNP copy number change and LOH in SS in the literature (http://www.ncbi.nlm.nih.gov/sites/entrez).

To address this issue we initially performed SNP microarray on DNA samples isolated from biopsies of skin lesions and peripheral blood mononuclear cells (PBMCs) of patients with SS by using Affymetrix GeneChip Mapping 10K Xba assay. Then we assessed gene-specific SNP copy number changes in same DNA samples with ABI TaqMan SNP genotyping assays to compare and verify the findings from Affymetrix 10K SNP microarray analysis. Finally, we conducted a literature review on LOH findings of patients with SS. Our findings are presented here in this paper.

## 2. Materials and Methods

### 2.1. Sample Selection and DNA Preparation

**Figure 1:**
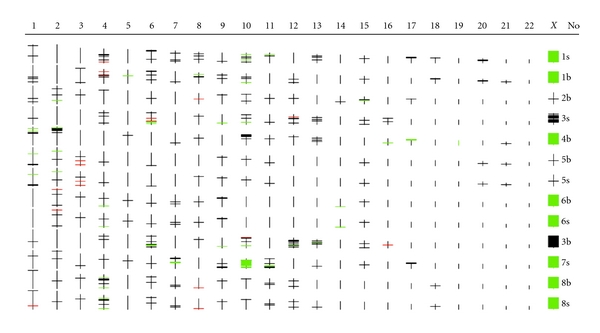
Illustration of a diagram of genomewide SNP homozygosity mapping in 8 SS cases (No. 1–8) detected with Affymetrix GeneChip Human Mapping 10K Array (http://www.affymetrix.com/products/arrays/specific/10k.affx) and analysed by using GOLF software (https://bioinformatics.cancerresearchuk.org/cazier01). Here each red-coloured line represents copy number gain of individual SNP, green-coloured line and block stand for SNP copy number loss, black-coloured line and block denote balanced/neutral SNP copy number or uniparental disomy. Similar SNP homozygosity profile is present in paired DNA samples from the biopsied skin lesion (S) and peripheral blood (B) isolated from individual patient with SS on the right end of the diagram. Frequent SNP homozygous changes (>3) are seen on almost all autosomes consistent with SNP copy number alterations shown in Figure 2. The remarkable changes on X chromosome were used as the internal quality control for the 10K SNP array experiment as green represents X chromosome copy number loss or monosomy indicating male sex, and black signifies balanced or disomy X chromosome denoting female.

**Figure 2:**
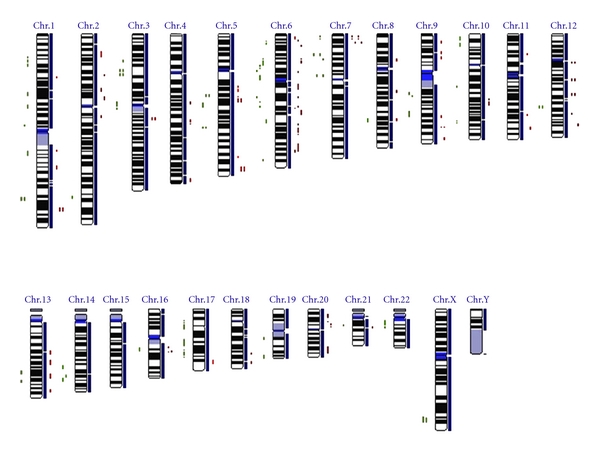
Illustration of an ideogram of genomewide SNP copy number changes in 8 SS cases identified by using Affymetrix GeneChip Human Mapping 10K Array as above and analysed with the IdeogramBrowser Software (http://www.informatik.uni-ulm.de/ni/staff/HKestler/ideo/doc.html). Here each red-coloured dot represents copy number loss of individual SNP and green-coloured dot stands for SNP copy number gain. Frequent SNP copy number changes at chromosomes 1, 2p, 3, 4q, 5q, 6, 7p, 8, 9, 10, 11, 12q, 13, 14, 16q, 17, and 20 are clearly visible.

Eleven patients with Sézary syndrome were selected for this study according to the World Health Organization and the European Organization for Research and Treatment of Cancer diagnosis criteria for CTCL [2]. Genomewide SNP analysis was performed on 8 of these SS cases using Affymetrix SNP microarray. Five of these 8 SS cases plus additional 3 cases were further assessed with SNP quantitative real-time PCR in this study. Briefly, a total of 16 DNA samples (13 samples for SNP microarray and 3 additional samples for SNP qPCR), which were taken prior to treatment, were extracted from both PBMCs and biopsied skin lesions from these SS cases (Figures 1 and 2). This was conducted by using QIAGEN Genomic-tip 100/G kit (QIAGEN Ltd, West Sussex, UK) according to the manufacturer's instruction (http://www1.qiagen.com/). DNA concentrations and purity were determined with the NanoDrop ND-1000 spectrophotometer (NanoDrop, Rockland, Del, USA). Local ethical approval for the sampling procedures was obtained previously [9–11, 18] and the use of the stored DNA samples for this study was approved by the Research Ethical Committee at Barts and Royal London School of Medicine and Dentistry, Queen Mary University of London.

### 2.2. Single Nucleotide Polymorphism Microarray Assay

Affymetrix SNP microarray analysis of 13 DNA samples including 5 paired PBMCs and skin biopsies from 8 SS patients (Figure 1) was conducted according to the standard GeneChip Mapping 10 K (V2.0) Xba Assay protocol (Affymetrix Inc., Santa Clara, Calif, USA). Briefly, 350 ng of DNA was digested with XbaI and ligation to the XbaI adaptor prior to PCR amplification (35 cycles) using AmpliTaq Gold with Buffer II (Applied Biosystems, Foster City, Calif, USA). Hybridised arrays were processed with an Affymetrix Fluidics Station 450 and fluorescence signals were detected using the Affymetrix GeneChip Scanner 3000. Signal intensity data was assessed with the GeneChip DNA analysis software (http://www.affymetrix.com/products/arrays/specific/10k.affx) based on a model algorithm to generate SNP calls [43] and subsequent bioinformative analysis was carried out using an in-house software called GOLF (http://www.bioinformatics.cancerresearchuk.org/cazier01) developed by Professor Young [44] and a publicly accessible software named IdeogramBrowser Software (http://www.informatik.uni-ulm.de/ni/staff/HKestler/ideo/doc.html).

### 2.3. SNP Quantitative Real-Time PCR Assay

**Figure 3:**
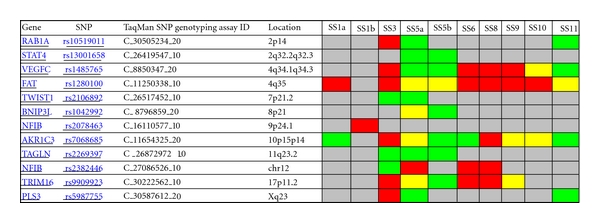
A summary of SNP copy number changes of 12 genes in 10 SS samples by using qPCR with ABI TaqMan SNP genotyping assay to verify the results of Affymetrix 10K SNP microarray analysis of SS in this study. Here red-coloured rectangular bar denotes SNP copy number loss against internal controls of B2M and GAPDH, green-coloured bar stands for SNP copy number gain, yellow-coloured bar represents balanced/neutral or normal SNP copy number, and gray-coloured bar indicates noninformative. Frequent SNP copy number losses of VEGFC, FAT, NFIB, and TRIM16 are clearly visible.

To verify the results of Affymetrix SNP microarray analysis of SS cases in this study, SNP qPCR assay was performed to test 10 DNA samples from 8 SS patients including 7 samples from 5 SS cases used in above SNP microarray and 3 samples from additional 3 SS cases (Figure 3). This experiment was carried out by means of an ABI Prism 7900 sequence detection system using the TaqMan PCR Master Mix with TaqMan SNP genotyping assays (http://www3.appliedbiosystems.com/applicationstechnolo-gies/real-timepcr/index.htm). These assays amplify 12 genes within the chromosomal regions showing frequent SNP copy number changes identified in this study (Figure 2). Additional 2 SNP genotyping assays amplifying beta-2-microglobulin (B2M, rs935885, C_12080829_10) and glyceraldehyde-3-phosphate dehydrogenase (GAPDH, rs3741918, C_27510362_10) were used as the internal quality controls. qPCR reactions contained 900 nmol primers, 200 nmol probes, 400 mM each dATP, dCTP and dGTP, 800 mM dUTP, 1 U Amplitaq Gold DNA polymerase, 0.2 U AmpErase uracil N-glycosylase (UNG), and 13 TaqMan buffer in a total volume of 25 *μ*L in 96-well microtiter plate (Applied Biosystems). After a decontamination step at 50°C, a two-step protocol was followed for 50 cycles: 95°C for 15 s and 61°C for 1 min. SNP qPCR data analysis was conducted as previously described [45] with ABI SNPbrowser Software (http://marketing.appliedbiosystems.com/mk/get/snpb_landing).

### 2.4. SNP Loss of Heterozygosity Expression Profiling

We have previously put forward a concept of functional copy number changes in cancer cells [46]. To further test our hypothesis in this study, we assume a SNP LOH gene cluster in SS is bioinformatically significant or functional if it is capable of separating all SS cases from normal control (NC) samples. This test was carried out through a data mining experiment by using a bioinformative method developed by us [46]. In this experiment, the effect of SNP LOH detected in this study on gene expression profiling in SS cases conducted in previous studies [23, 24, 47] was tested as before [46]. The procedures were briefly described as follows. We initially created gene lists from 9 chromosomal regions showing frequent LOH (>3 per region) at SNP level seen in SS (Table 1) by using IdeogramBrowser Software and UCSC Human Gene Sorter (http://www.genome.ucsc.edu/). Then we imported these 9 gene-lists into GeneSpring software version 7 (http://www.sigenetics.com/), which contained a genome/raw data of gene expression profile from 6 SS patients who were treated with photopheresis therapy and 2 healthy individuals generated by using Affymetrix GeneChip U133A (http://www.affymetrix.com/products/arrays/specific/hgu133av2.affx). Of 6 SS cases analysed with Affymetrix gene expression microarray the 3 cases were also tested by above Affymetrix SNP microarray. Finally, we analysed the selected 9 gene lists by using the clustering tool of GeneSpring to generate gene tree and condition tree, a supervised clustering, which enables direct visualisation of sample congregation and separation [46].

### 2.5. Comparison with Published Data on LOH at Microsatellite Loci in SS

The genomic region where SNPs span on Affymetrix 10K GeneChip used in this study is equivalent to that covered by 400 microsatellite (MS) loci (http://www.affymetrix.com/products/arrays/specific/10k.affx). There have been several publications describing LOH at MS loci on limited chromosomes in CTCL including SS [9, 15–19, 48]. To compare previous findings with this study, we reanalysed previous published data on MS LOH at chromosomes 1 and 10q through fine mapping of each MS locus, which was linked to individual gene and SNP by using NCBI ENTREZ GENE and ENTREZ SNP database (http://www.ncbi.nlm.nih.gov/).

## 3. Results

### 3.1. SNP Microarray

In this study, paired DNA samples from PBMCs and skin lesions from 5 of 8 SS cases were analysed with Affymetrix 10 K SNP microarray, which showed similar SNP homozygous changes between the paired samples indicating they are coming from the same individual. There was a difference in SNP fingerprint among 8 different SS cases signifying the absence of sample contamination. As Figure 1 shows, frequent homozygous changes (>3 per chromosome) including gain, loss, and uniparental disomy (UPD) were seen to distribute throughout almost an entire genome except of chromosomes 14, 19 and 22.

As Figure 2 shows there were frequent SNP copy number changes (>3 per chromosome) at chromosomes 1, 2p, 3, 4q, 5q, 6, 7p, 8, 9, 10, 11, 12q, 13, 14, 16q, 17, and 20, which are not only consistent with above homozygous changes but also in line with previous CGH findings in SS [10]. 

In this study, a weak and/or smear band of TCR gene was detected in skin DNA samples, while a strong monoclonal TCR gene band was seen in PBMC DNA samples (data not shown), suggesting that only a few tumour cells were present in skin samples and sample contamination was negligible. Overall, a frequent LOH was noted in 5 paired SS cases at 9 chromosomal regions including 1p36p33, 2q21q24, 8q22q24, 9q31q34, 10p11q26, 11q21q25, 13q11q12, 16q21q23, and 17p13q11 (Table 1), which are to some extent similar to previous reports on LOH at certain chromosome regions [9, 15–19].

### 3.2. SNP qPCR Assay

To further confirm SNP microarray findings, SNP qPCR was utilised to test DNA samples from 5 of 8 SS cases (case number 1, 3, 5, 6 and 8) analysed with Affymetrix 10K SNP microarray using ABI TaqMan SNP genotyping assay (63%) and 3 additional cases. As Figure 3 shows, frequent SNP copy number loss (>2 cases) at the FAT gene (rs1280100) at 4q35 was observed in 6 of 8 SS cases (number 1, 3, 6, 8, 9, and 10) (75%), followed by VEGFC (rs1485765) at 4q34.1q34.3 (cases  3, 6, 8, and 9) (50%), NFIB (rs2382446) at chromosome 12 (cases  5, 6 and 8) (38%), and TRIM16 (rs9909923) at 17p11.2 (cases  3, 6, and 8) (38%). While frequent SNP copy number gain (>2 cases) was also noted at the AKR1C3 gene (rs7068685, 50%), VEGFC (rs1485765, 38%), and TAGLN (rs2269397, 38%). 

SNP copy number changes were most frequent present in SS case  3, which showed losses of genes of RAB1A (rs10519011, 2p14), VEGFC, **FAT**, AKR1C3, TRIM16, and PLS3 (rs5987755, Xq23) as well as gains of genes of TAGLN (rs2269397, 11q23.2) and NFIB. This was followed by case  5, which had gains of RAB1A, STAT4 (rs13001658, 2q32.2q32.3), VEGFC, TWIST1 (rs2106892, 7p21.2), TAGLN, and PLS3, and loss of NFIB. Case  6 revealed losses of VEGFC, **FAT**, NFIB and TRIM16, and gain of AKR1C3; case  8 demonstrated losses of VEGFC, **FAT**, AKR1C3, NFIB, and TRIM16 (Figure 3). Overall these SNP copy number changes, detected by using qPCR with ABI TaqMan SNP genotyping assay, were consistent with those identified by using Affymetrix 10K SNP microarray as shown in Figure 2.

### 3.3. SNP LOH Expression Profiling

**Figure 4:**
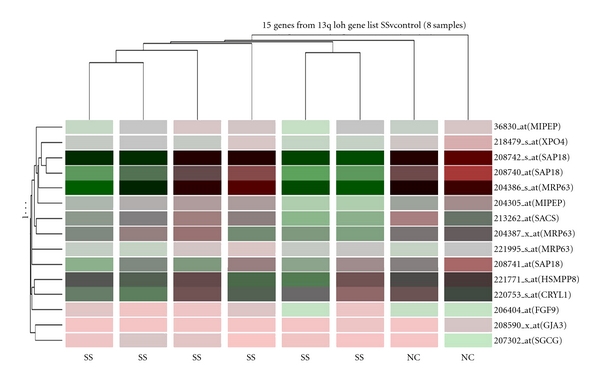
Illustration of a heatmap of gene expression profile of SNP LOH at 13q11q12 gene cluster (15 genes). Here each column represents one test sample and each coloured rectangular bar signifies each individual gene. The colouration of each bar indicates the expression level of individual gene, which has been reported previously [23, 24, 47] and is not the focus of this study. The separation of 6 Sézary syndrome cases (SS, left and middle) from 2 normal controls (NC, right) is clearly visible and two test groups are different.

The impact of SNP LOH gene cluster on gene expression pattern was further tested in this study. As shown in Figure 4, the clusters of 2 NCs appeared at the right end of the heatmap and the clusters of 6 SS cases were present in the middle and at the left end of the heatmap. This clearly showed that two groups were separated and different. Over the 9 SNP LOH gene lists analysed in this study, 3 (33%) showed gene expression patterns of the separation of 6 SS cases from 2 NCs (Table 1). This included SNP LOH gene clusters at 9q31q34 (40 genes), 10p11q26 (111 genes), and 13q11q12 (15 genes) (Figure 4). Each individual gene in these 3 gene clusters is listed in Table 2. However, the remaining 6 SNP LOH gene lists revealed a mixed gene expression pattern without the separation of SS from NCs (Table 1).

### 3.4. Refining Previous SS LOH Data and Comparing with This Study

**Figure 5:**
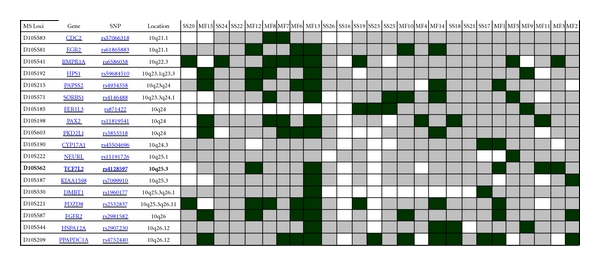
A summary of reanalysis of previously published LOH at 18 microsatellite loci on 10q21q26 in SS and MF [19] with this study, in which each microsatellite locus is finely mapped and linked to individual gene and SNP via NCBI ENTREZ GENE and ENTREZ SNP database (http://www.ncbi.nlm.nih.gov/). Here black bar represents LOH, gray bar stands for noninformative, and white bar indicates microsatellite marker that is not available. One SNP within microsatellite locus D10S562 (bold) is also present in the SNP LOH cluster at 10p11q26 in this study (Table 1).

Previous studies have shown LOH on several chromosomes including chromosomes 1 and 10q [9, 15–19, 48]. The 10K SNP microarray gene chip used in this study is equivalent to the genetic distance covered by 400 MS. This allows us to compare SNP LOH identified in this study with MS LOH described in those previous studies. Frequent LOH at MS loci D1S247 (rs11372930) at 1p36 and D10S562 (rs4128597) at 10q25.3 (Figure 5) reported in previous studies [9, 18, 19] were seen in this study.

## 4. Discussion

This integrated genomic study has for the first time revealed genomewide SNP copy number change and LOH as well as SNP LOH gene expression profiling in SS cases. Although the number of SS cases tested in this study was small, the patterns of SNP copy number change and LOH identified in SS cases are generally in line with previous metaphase-/array-CGH [6–14] and MS LOH studies [9, 15, 17–19, 48], suggesting the high efficiency and reliability of the methods used. In addition some novel findings emerged. This includes the presence of SNP copy number loss at the FAT gene (4q35) in most SS cases and the separation of all SS cases from NC samples by SNP LOH gene clusters at chromosome regions of 9q31q34, 10p11q26, and 13q11q12. Although all of these results will need to be validated in larger cohorts of samples from SS patients in the future, they do provide some intriguing information for our current understanding of the molecular pathogenesis of this type of lymphoma and suggest the possibility of presence of functional SNP LOH in SS tumour cells at least from the point of view of bioinformatics. 

Sézary syndrome is a rare form of CTCL with an aggressive clinical course and it also likely represents the leukaemic phase of mycosis fungoides [1, 2]. Like any other advanced stage of malignancies, SS accumulates a variety of genetic and epigenetic events including alterations of tumour suppressor genes, oncogenes, and some key house-keeping genes [18, 27, 28, 49]. Previous studies on SS have shown complex genetic aberrations affecting almost all chromosomes with copy number losses being the dominant abnormalities, which mainly involve chromosomes 1p, 6q, 9, 10, 13q, and 17p [6–14] and the presence of LOH at MS loci on chromosomes 1p, 9p, 10q, and 17p in tumour cells [9, 15–19, 48]. In addition, downregulation of several genes including MXI1 (10q24q25) and upregulation of genes such as TWIST1 (7p21.2) and PLS3 (Xq23) have also been described in this malignancy [20–26]. Similar results were obtained in this study, which showed SNP copy number change on chromosomes 1, 2p, 3, 4q, 5q, 6, 7p, 8, 9, 10, 11, 12q, 13, 14, 16q, 17, and 20, and LOH at 1p, 2q, 8q, 9q, 10, 11q, 13q, 16q, and 17 as well as SNP copy number gains of TWIST1 and PLS3 in 2 SS cases, further indicating the high efficiency and reliability of the integrated genomic experiments conducted in this study. 

Although there are different definitions for loss of heterozygosity in literature, LOH is generally defined as a locus or loci at which a deletion or other process has converted the locus from heterozygosity to homozygosity or hemizygosity. In terms of cancer, LOH signifies that in tumour cells carrying a mutated allele of a tumour suppressor gene, the gene becomes fully inactivated when the cell loses a large part of the chromosome carrying the wild-type allele (http://www.ncbi.nlm.nih.gov/cancerchromosomes). LOH is traditionally used as a molecular tool to identify a tumour-suppressor gene but it also represents one of the most common molecular features of cancer cells [47, 50–52]. Previous studies have shown LOH at MS loci on chromosomes 1p, 9p, 10q, 12q, 13q, 17p, and 19 in CTCL including SS [9, 15–19, 48]. This was supported by previous conventional cytogenetics and metaphase and array-based CGH studies, which revealed DNA copy number losses at these chromosomal regions [6–14, 48]. In this study LOH at SNP level on 9 chromosomal regions (1p, 2q, 8q, 9q, 10, 11q, 13q, 16q, and 17) was identified in SS cases by using Affymetrix 10 K GeneChip, which covers approximately 400 MS loci. There was an overlap between this and previous studies on LOH at chromosome 1p (D1S247) and 10q (D10S562). Previous studies also suggested LOH at 1p and 10q associated with late stage CTCL [15–17, 19, 48]. In addition, copy number losses were described in these 9 chromosomal regions by using molecular cytogenetic techniques as discussed above. Furthermore, these findings were subsequently validated by extensive SNP qPCR test in this study. Taking together all of these results suggest that some of these SNP LOH and copy number changes are likely to be associated with the pathogenesis of SS.

The FAT gene, which is expressed at high levels in a number of foetal epithelia, is an ortholog of the Drosophila fat gene. FAT encodes a tumour suppressor essential for controlling cell proliferation during Drosophila development. Its product is a member of the cadherin superfamily, a group of integral membrane proteins characterised by the presence of 34 tandem cadherin-type repeats as well as five epidermal growth factor-like repeats and one laminin A-G domain with possible functions as an adhesion molecule and/or signalling receptor, which are likely to be associated with developmental processes and cell communication (http://www.ncbi.nlm.nih.gov/gene/2195). The FAT gene is located on human chromosome 4q35. Previous molecular cytogenetic studies have revealed copy number losses at this chromosomal region in a variety of cancers (http://www.progenetix.org/cgi-bin/pgCasesMatrixPlotter.cgi). Furthermore deletion/LOH and absent expression of FAT have also been found in oral cancer [53] and human astrocytic tumours [54]. Intriguingly, in this study SNP copy number loss of FAT was observed in three-quarter of SS cases and reduced gene expression of FAT was also noted in most cutaneous squamous cell carcinoma cases (data not shown) no spare SS RNA samples are available for qPCR testing. These results further indicate the tumour suppressor role of FAT and also suggest that loss of FAT is relevant to SS pathology.

Interindividual copy number variation/polymorphism (CNV/P) is thought to be the manifestation of a considerable and unanticipated plasticity of the human genome. CNV/P constitutes a major source of interindividual genetic variation that could explain variable penetrance of inherited diseases and variation in the phenotypic expression of aneuploidies and sporadic traits [55, 56]. There is increasing evidence showing de novo CNV/P as a major cause of mental retardation and several other complex disorders [38–40, 43, 57]. Cytogenetically CNV/P or copy number changes (CNCs) have long been described in cancer cells (http://www.cgap.nci.nih.gov/Chromosomes/Mitelman, http://www.progenetix.net/). As discussed above SS has been found with frequent CNCs at several chromosomal regions [6–13]. Some of these CNCs may have a net effect on gene expression as previous expression profiling studies on SS have revealed up- and downregulations of several genes, which are mapped within the chromosomal regions harbouring these CNCs [20–26]. From, bioinformatic point of view these CNCs may be functional. Whilst the remaining CNCs (CNV/P) may not have any net effect at all and are therefore nonfunctional. To test this hypothesis we developed a simple bioinformatics strategy through the analysis of the expression profiling of CNCs gene clusters commonly presented in SS to see if SS cases can be separated from NCs. We found separated expression patterns in 4 of 17 CNCs gene clusters [46]. In this study, we expanded the scope of our bioinformatics experiment by examining the impact of common SNP LOH gene clusters identified in SS cases with Affymetrix 10 K SNP microarray on gene expression patterns in SS cases detected using gene expression microarray with Affymetrix GeneChip U133A and dedicated software. There was a separation of all 6 SS cases from 2 NCs by 3 of 9 SNP LOH gene clusters. This includes 9q31q34, 10p11q26, and 13q11q12, which lie within the chromosomal regions showing frequent LOH and/or DNA copy number loss in SS. In addition, decreased expression of MXI1 in SNP LOH gene cluster at 10p11q26 has been described in SS previously [22]. All of these findings suggest that the 3 SNP LOH gene clusters may be functional in terms of bioinformatics and relevant to SS pathology. There are also a number of genes in these 3 SNP LOH gene lists which have not been described in SS before. Further functional studies, including proteomic analysis, are therefore necessary to elucidate the oncogenic role of these genes in the initiation and progression of SS and other types of CTCL.

## Figures and Tables

**Table 1:** A summary of gene expression profile of LOH gene clusters.

Chromosomal regions with LOH detected in this study	Number of genes	Separation of SS from NC*
1p36p33	80	N
2q21q24	9	N
8q22q24	15	N
9q31q34	40	P
10p11q26	111	P
11q21q25	205	N
13q11q12	15	P
16q21q23	74	N
17p13q11	198	N

*N: no separation between SS and NC, P: presence of separation between SS and NC.

**Table 2:** A detailed gene list of the 3 LOH gene clusters separating SS from NC

LOH gene clusters	Gene name	Common	Description
9q31q34	221294_at	GPR21	G-protein-coupled receptor 21
	221085_at	TNFSF15	Tumor necrosis factor (ligand) superfamily, member 15
	220935_s_at	CDK5RAP2	CDK5 regulatory subunit associated protein 2
	220300_at	RGS3	Regulator of G-protein signalling 3
	220202_s_at	MNAB	Membrane-associated nucleic acid binding protein
	220201_at	MNAB	Membrane-associated nucleic acid binding protein
	219884_at	LHX6	LIM homeobox protein 6
	218941_at	FBXW2	F-box and WD-40 domain protein 2
	218489_s_at	ALAD	Aminolevulinate, delta-, dehydratase
	218487_at	ALAD	Aminolevulinate, delta-, dehydratase
	218160_at	NDUFA8	NADH dehydrogenase (ubiquinone) 1 alpha subcomplex, 8, 19 kDa
	215813_s_at	PTGS1	Prostaglandin-endoperoxide synthase 1 (prostaglandin G/H synthase and cyclooxygenase)
	214465_at	ORM2	Orosomucoid 2
	214425_at	AMBP	Alpha-1-microglobulin/bikunin precursor
	214040_s_at	GSN	Gelsolin (amyloidosis, Finnish type)
	211503_s_at	RAB14	RAB14, member RAS oncogene family
	210825_s_at	STOM	Stomatin
	210824_at	STOM	Stomatin
	209162_s_at	PRPF4	PRP4 pre-mRNA processing factor 4 homolog (yeast)
	209161_at	PRPF4	PRP4 pre-mRNA processing factor 4 homolog (yeast)
	208828_at	POLE3	Polymerase (DNA directed), epsilon 3 (p17 subunit)
	208737_at	ATP6V1G1	ATPase, H+ transporting, lysosomal 13 kDa, V1 subunit G isoform 1
	205599_at	TRAF1	TNF receptor-associated factor 1
	205500_at	C5	Complement component 5
	205477_s_at	AMBP	Alpha-1-microglobulin/bikunin precursor
	205128_x_at	PTGS1	Prostaglandin-endoperoxide synthase 1 (prostaglandin G/H synthase and cyclooxygenase)
	205127_at	PTGS1	Prostaglandin-endoperoxide synthase 1 (prostaglandin G/H synthase and cyclooxygenase)
	205041_s_at	ORM1	Orosomucoid 1
	205040_at	ORM1	Orosomucoid 1
	204449_at	PDCL	Phosducin-like
	204448_s_at	PDCL	Phosducin-like
	203971_at	SLC31A1	Solute carrier family 31 (copper transporters), member 1
	203823_at	RGS3	Regulator of G-protein signalling 3
	203447_at	PSMD5	Proteasome (prosome, macropain) 26S subunit, non-ATPase, 5
	201062_at	STOM	Stomatin
	201061_s_at	STOM	Stomatin
	201060_x_at	STOM	Stomatin
	200928_s_at	RAB14	RAB14, member RAS oncogene family
	200927_s_at	RAB14	RAB14, member RAS oncogene family
	200696_s_at	GSN	Gelsolin (amyloidosis, Finnish type)

10p11.21q26.3	32094_at	CHST3	Carbohydrate (chondroitin 6) sulfotransferase 3
	222072_at	ADD3	Homo sapiens clone DT1P1A11 mRNA, CAG repeat region
	221136_at	GDF2	Growth differentiation factor 2
	219963_at	DUSP13	Dual specificity phosphatase 13
	219957_at	RUFY2	RUN and FYVE domain containing 2
	219543_at	MAWBP	MAWD binding protein
	219509_at	MYOZ1	Myozenin 1
	218878_s_at	SIRT1	Sirtuin (silent mating type information regulation 2 homolog) 1 (*S. cerevisiae*)
	218871_x_at	GALNACT-2	Chondroitin sulfate GalNAcT-2
	218445_at	H2AFY2	H2A histone family, member Y2
	218249_at	ZDHHC6	Zinc finger, DHHC domain containing 6
	218046_s_at	MRPS16	Mitochondrial ribosomal protein S16
	218006_s_at	ZNF22	Zinc finger protein 22 (KOX 15)
	218005_at	ZNF22	Zinc finger protein 22 (KOX 15)
	216903_s_at	CBARA1	Calcium binding atopy-related autoantigen 1
	216345_at	KIAA0913	KIAA0913 protein
	216037_x_at	TCF7L2	Transcription factor 7-like 2 (T-cell specific, HMG-box)
	216035_x_at	TCF7L2	Transcription factor 7-like 2 (T-cell specific, HMG-box)
	214878_at	ZNF37A	Zinc finger protein 37a (KOX 21)
	214617_at	PRF1	Perforin 1 (pore forming protein)
	214508_x_at	CREM	cAMP responsive element modulator
	214366_s_at	ALOX5	Arachidonate 5-lipoxygenase
	214348_at	TACR2	Tachykinin receptor 2
	214338_at	DNAJB12	DnaJ (Hsp40) homolog, subfamily B, member 12
	214322_at	CAMK2G	Calcium/calmodulin-dependent protein kinase (CaM kinase) II gamma
	214136_at	NUDT13	Nudix (nucleoside diphosphate linked moiety X)-type motif 13
	213952_s_at	ALOX5	Arachidonate 5-lipoxygenase
	213647_at	DNA2L	DNA2 DNA replication helicase 2-like (yeast)
	212894_at	SUPV3L1	Suppressor of var1, 3-like 1 (*S. cerevisiae*)
	212762_s_at	TCF7L2	Transcription factor 7-like 2 (T-cell specific, HMG-box)
	212761_at	TCF7L2	Transcription factor 7-like 2 (T-cell specific, HMG-box)
	212759_s_at	TCF7L2	Transcription factor 7-like 2 (T-cell specific, HMG-box)
	212757_s_at	CAMK2G	Calcium/calmodulin-dependent protein kinase (CaM kinase) II gamma
	212669_at	CAMK2G	Calcium/calmodulin-dependent protein kinase (CaM kinase) II gamma
	212594_at	PDCD4	Programmed cell death 4 (neoplastic transformation inhibitor)
	212593_s_at	PDCD4	Programmed cell death 4 (neoplastic transformation inhibitor)
	212359_s_at	KIAA0913	KIAA0913 protein
	212322_at	SGPL1	Sphingosine-1-phosphate lyase 1
	212321_at	SGPL1	Sphingosine-1-phosphate lyase 1
	211809_x_at	COL13A1	Collagen, type XIII, alpha 1
	211668_s_at	PLAU	plasminogen activator, urokinase
	211343_s_at	COL13A1	Collagen, type XIII, alpha 1
	210956_at	PPYR1	Pancreatic polypeptide receptor 1
	210671_x_at	MAPK8	Mitogen-activated protein kinase 8
	210588_x_at	HNRPH3	Heterogeneous nuclear ribonucleoprotein H3 (2H9)
	210477_x_at	MAPK8	Mitogen-activated protein kinase 8
	210318_at	RBP3	Retinol binding protein 3, interstitial
	210171_s_at	CREM	cAMP responsive element modulator
	210110_x_at	HNRPH3	Heterogeneous nuclear ribonucleoprotein H3 (2H9)
	209910_at	SLC25A16	Solute carrier family 25 (mitochondrial carrier; Graves disease autoantigen), member 16
	209869_at	ADRA2A	Adrenergic, alpha-2A-, receptor
	209860_s_at	ANXA7	Annexin A7
	209834_at	CHST3	Carbohydrate (chondroitin 6) sulfotransferase 3
	209817_at	PPP3CB	Protein phosphatase 3 (formerly 2B), catalytic subunit, beta isoform (calcineurin A beta)
	209538_at	ZNF32	Zinc finger protein 32 (KOX 30)
	209457_at	DUSP5	Dual specificity phosphatase 5
	209045_at	XPNPEP1	X-prolyl aminopeptidase (aminopeptidase P) 1, soluble
	208990_s_at	HNRPH3	Heterogeneous nuclear ribonucleoprotein H3 (2H9)
	208770_s_at	EIF4EBP2	Eukaryotic translation initiation factor 4E binding protein 2
	208769_at	EIF4EBP2	Eukaryotic translation initiation factor 4E binding protein 2
	208535_x_at	COL13A1	Collagen, type XIII, alpha 1
	208453_s_at	XPNPEP1	X-prolyl aminopeptidase (aminopeptidase P) 1, soluble
	208381_s_at	SGPL1	Sphingosine-1-phosphate lyase 1
	208252_s_at	CHST3	Carbohydrate (chondroitin 6) sulfotransferase 3
	208152_s_at	DDX21	DEAD/H (Asp-Glu-Ala-Asp/His) box polypeptide 21
	208095_s_at	CAMK2G	Calcium/calmodulin-dependent protein kinase (CaM kinase) II gamma
	207965_at	NEUROG3	Neurogenin 3
	207630_s_at	CREM	cAMP responsive element modulator
	207543_s_at	P4HA1	Procollagen-proline, 2-oxoglutarate 4-dioxygenase (proline 4-hydroxylase), alpha polypeptide I
	207347_at	ERCC6	Excision repair cross-complementing rodent repair deficiency, complementation group 6
	207127_s_at	HNRPH3	Heterogeneous nuclear ribonucleoprotein H3 (2H9)
	206747_at	KIAA0514	KIAA0514 gene product
	206261_at	ZNF239	Zinc finger protein 239
	206159_at	GDF10	Growth differentiation factor 10
	206010_at	HABP2	Hyaluronan binding protein 2
	205882_x_at	ADD3	Adducin 3 (gamma)
	205879_x_at	RET	Ret proto-oncogene (multiple endocrine neoplasia and medullary thyroid carcinoma 1, Hirschsprung disease)
	205479_s_at	PLAU	Plasminogen activator, urokinase
	204446_s_at	ALOX5	Arachidonate 5-lipoxygenase
	204445_s_at	ALOX5	Arachidonate 5-lipoxygenase
	204120_s_at	ADK	Adenosine kinase
	204119_s_at	ADK	Adenosine kinase
	203916_at	NDST2	N-deacetylase/N-sulfotransferase (heparan glucosaminyl) 2
	203666_at	CXCL12	Chemokine (C-X-C motif) ligand 12 (stromal cell-derived factor 1)
	203187_at	DOCK1	Dedicator of cyto-kinesis 1
	203079_s_at	CUL2	Cullin 2
	203078_at	CUL2	Cullin 2
	203074_at	ANXA8	Annexin A8
	202867_s_at	DNAJB12	DnaJ (Hsp40) homolog, subfamily B, member 12
	202866_at	DNAJB12	DnaJ (Hsp40) homolog, subfamily B, member 12
	202865_at	DNAJB12	DnaJ (Hsp40) homolog, subfamily B, member 12
	202777_at	SHOC2	soc-2 suppressor of clear homolog (C. elegans)
	202731_at	PDCD4	Programmed cell death 4 (neoplastic transformation inhibitor)
	202730_s_at	PDCD4	Programmed cell death 4 (neoplastic transformation inhibitor)
	202524_s_at	SPOCK2	Sparc/osteonectin, cwcv and kazal-like domains proteoglycan (testican) 2
	202523_s_at	SPOCK2	Sparc/osteonectin, cwcv and kazal-like domains proteoglycan (testican) 2
	202432_at	PPP3CB	Protein phosphatase 3 (formerly 2B), catalytic subunit, beta isoform (calcineurin A beta)
	202364_at	MXI1	MAX interacting protein 1
	202361_at	SEC24C	SEC24 related gene family, member C (*S. cerevisiae*)
	201859_at	PRG1	Proteoglycan 1, secretory granule
	201858_s_at	PRG1	Proteoglycan 1, secretory granule
	201753_s_at	ADD3	Adducin 3 (gamma)
	201752_s_at	ADD3	Adducin 3 (gamma)
	201376_s_at	HNRPF	Heterogeneous nuclear ribonucleoprotein F
	201366_at	ANXA7	Annexin A7
	201034_at	ADD3	Adducin 3 (gamma)
	200931_s_at	VCL	Vinculin
	200930_s_at	VCL	Vinculin
	200871_s_at	PSAP	Prosaposin (variant Gaucher disease and variant metachromatic leukodystrophy)
	200866_s_at	PSAP	Prosaposin (variant Gaucher disease and variant metachromatic leukodystrophy)
	200697_at	HK1	Hexokinase 1

13q11q12	36830_at	MIPEP	Mitochondrial intermediate peptidase
	221995_s_at	MRP63	Mitochondrial ribosomal protein 63
	221771_s_at	HSMPP8	M-phase phosphoprotein, mpp8
	220753_s_at	CRYL1	Crystallin, lambda 1
	218479_s_at	XPO4	Exportin 4
	213262_at	SACS	Spastic ataxia of Charlevoix-Saguenay (sacsin)
	208742_s_at	SAP18	sin3-associated polypeptide, 18 kDa
	208741_at	SAP18	sin3-associated polypeptide, 18 kDa
	208740_at	SAP18	sin3-associated polypeptide, 18 kDa
	208590_x_at	GJA3	Gap junction protein, alpha 3, 46 kDa (connexin 46)
	207302_at	SGCG	Sarcoglycan, gamma (35 kDa dystrophin-associated glycoprotein)
	206404_at	FGF9	Fibroblast growth factor 9 (glia-activating factor)
	204387_x_at	MRP63	Mitochondrial ribosomal protein 63
	204386_s_at	MRP63	Mitochondrial ribosomal protein 63
	204305_at	MIPEP	Mitochondrial intermediate peptidase
